# Supplementary material for: Feline infectious peritonitis epizootic caused by a recombinant coronavirus
Source: Nature. 2025 Jul 9;645(8079):228–34. doi: 10.1038/s41586-025-09340-0 (PMC12408369; doi:10.1038/s41586-025-09340-0)
Supplement: Supplementary file 1 — Supplementary Methods relating to Extended Data Fig. 6, Supplementary Tables 1–26 and Supplementary References. [file 41586_2025_9340_MOESM1_ESM.pdf]

nature portfolio

https://doi.org/10.1038/s41586-025-09340-0

Supplementary information

Feline infectious peritonitis epizootic caused by a recombinant coronavirus

In the format provided by the authors and unedited

Feline infectious peritonitis epizootic caused by a recombinant coronavirus

Charalampos Attipa[1,2,3◇*], Amanda S Warr[2◇*], Demetris Epaminondas[4], Marie O'Shea[2], Andrew J Hanton[2], Sarah Fletcher[2], Alexandra Malbon[13], Maria Lyraki[5], Rachael Hammond[1], Alexandros Hardas[6], Antria Zanti[3], Stavroula Loukaidou[3], Michaela Gentil[7], Danielle Gunn-Moore[1], Samantha J Lycett[2], Stella Mazeri[2], Christine Tait-Burkard[2*]

[1]Royal (Dick) School of Veterinary Studies, University of Edinburgh, Easter Bush, Midlothian, United Kingdom.

[2]The Roslin Institute, Royal (Dick) School of Veterinary Studies, University of Edinburgh, Easter Bush, Midlothian, United Kingdom.

[3]Vet Dia Gnosis Ltd, Limassol, Cyprus.

[4]Veterinary Services, Ministry of Agriculture, Natural Resources and Environment, Nicosia, Cyprus.

[5]Plakentia Veterinary Clinic, Athens, Greece.

[6]Department of Pathobiology and Population Sciences, Royal Veterinary College, Hatfield, Hertfordshire.

[7]Laboklin GmbH and Co KG, Bad Kissingen, Germany.

[◇]These authors contributed equally to this work.

*Correspondence to:

Email: christine.burkard@roslin.ed.ac.uk

Guide to supplementary information

Supplementary method relating to extended data figure 6 ... 1

Epidemiological metadata FIP outbreak Cyprus (relate to figure 1)
Supplementary table 1 ... 2
Supplementary table 2 ... 2
Supplementary table 3 ... 2
Supplementary table 4 ... 2
Supplementary table 5 ... 2
Supplementary table 6 ... 3

Epidemiological metadata RNA samples (relate to figure 1)
Supplementary table 7 ... 3
Supplementary table 8 ... 3
Supplementary table 9 ... 3
Supplementary table 10 ... 3
Supplementary table 11 ... 4
Supplementary table 12 ... 4

Epidemiological metadata spike sequenced samples (relate to figure 1)
Supplementary table 13 ... 4
Supplementary table 14 ... 4
Supplementary table 15 ... 5
Supplementary table 16 ... 5
Supplementary table 17 ... 5
Supplementary table 18 ... 5

Tiled amplicon sequencing primers (preliminary and final) (relate to methods)
Supplementary table 19 ... 6
Supplementary table 20 ... 8

Recombination analysis statistics (relate to figure 3)
Supplementary table 21 ... 11
Supplementary table 22 ... 11

Key amino acid locus analysis FECV/FIPV (relates to figure 4)
Supplementary table 23 ... 12
Supplementary table 24 ... 12
Supplementary table 25 ... 13

Reposited whole genome sequences accession numbers
Supplementary table 26 ... 14

References ... 15

Supplementary method relating to extended data figure 6

Bayesian time-resolved phylogenetic trees were estimated using BEAST 1.10.4[1] using the TN93 (Tamura-Nei 93) nucleotide substitution model with a four-category gamma distribution model of site-specific rate variation, a strict clock, the constant population size coalescent tree prior. For this dataset two independent Markov Chain Monte Carol (MCMC) chains were run, each chain consisted of 10,000,000 steps, was sampled every 1,000 steps, and the first 10% of samples were discarded as burn-in. The MCMC settings resulted in a post-burnin effective sample size of at least 200 (which is the accepted standard in BEAST analyses). The two independent chains were further down-sampled by a factor of 18 and combined to make 1,002 posterior trees, which were re-used in BEAST (as empirical trees) along with an asymmetric discrete trait model[2] for Place (5 states) with BSSVS (Bayesian Stochastic Search Variable Selection) and a homogenous Brownian motion diffusion model[3] for latitude and longitude coordinates to enable phylogeographic mapping. The resulting posterior trees from the mapping were summarized to a single Maximum Clade Credibility tree (MCC tree) using TreeAnnotator. The MCC tree was displayed using R-package ape[4] and custom R-scripts, including the addition of the spike deletion information on the tips of the tree.

Supplementary table 1

FIP outbreak Cyprus Jan 2023-Jun 2024; case distribution – sex

Sex	n	Total	Percentage
**Female**	65	215	33.02
**Male**	137	215	66.98

Supplementary table 2

FIP outbreak Cyprus Jan 2023-Jun 2024; case distribution – Age stats (years); the age of 51 cases are missing

	Mean_age	Median_age	Min_age	Max_age
**Jan 23- Mar-24**	**4.6059**	3	1	19
**Jan 23- Sep-23**	**4.4127**	3	1	16
**Oct-23-Mar-24**	**5.5423**	4.25	1	19
**Apr-24-Jun-24**	**5.1250**	3.7	0.6	13.3

Supplementary table 3

FIP outbreak Cyprus Jan 2023 -Jun 2024; case distribution – FIP form

Form	n	Total	Percentage
**Non-effusive**	22	215	10.23
**Neurological**	56	215	26.05
**Effusive**	137	215	63.72

Supplementary table 4

FIP outbreak Cyprus Jan 2023 -Sep 2023; case distribution – FIP form

Form	n	Total	Percentage
**Non-effusive**	4	167	2.4
**Neurological**	48	167	28.74
**Effusive**	115	167	68.86

Supplementary table 5

A) FIP outbreak Cyprus Oct 2023 -Mar 2024; case distribution – FIP form

Form	n	Total	Percentage
**Non-effusive**	12	34	35.29
**Neurological**	8	34	23.53
**Effusive**	14	34	41.18

B) FIP outbreak Cyprus Apr 2024 -Jun 2024; case distribution – FIP form

Form	n	Total	Percentage
**Non-effusive**	6	14	42.86
**Neurological**	0	14	0
**Effusive**	8	14	57.14

Supplementary table 6

FIP outbreak Cyprus Jan 2023-Mar 2024; case distribution – District

District	n	Total	Percentage
**Nicosia**	58	215	26.98
**Famagusta**	52	215	24.19
**Larnaca**	41	215	19.07
**Limassol**	34	215	15.81
**Paphos**	30	215	13.95

Supplementary table 7

RNA samples for sequencing; case distribution – Sex

Sex	n	Total	Percentage
**Female**	54	163	33.13
**Male**	109	163	66.87

Supplementary table 8

RNA samples for sequencing; case distribution – Age stats (years). The ages of 38 cats are missing.

Mean_age	Median_age	Min_age	Max_age
4.21	3	1	16

Supplementary table 9

RNA samples for sequencing; case distribution – FIP form

Form	n	Total	Percentage
**Non-effusive**	6	163	3.68
**Neurological**	42	163	25.77
**Effusive**	113	163	70.55

Supplementary table 10

RNA samples for sequencing; case distribution – District

District	n	Total	Percentage
**Nicosia**	46	163	28.22
**Famagusta**	46	163	28.22
**Larnaca**	31	163	19.02
**Limassol**	21	163	12.88
**Paphos**	17	163	10.43
**UK**	2	163	1.23

Supplementary table 11

RNA samples for sequencing; case distribution – Collection dates

District	n	Total	Percentage
**October-21**	1	163	0.61
**November-21**	1	163	0.61
**December-21**	1	163	0.61
**February-22**	1	163	0.61
**March-22**	1	163	0.61
**August-22**	1	163	0.61
**December-22**	1	163	0.61
**January-23**	7	163	4.29
**February-23**	23	163	14.11
**March-23**	36	163	22.09
**April-23**	19	163	11.66
**September-23**	28	163	17.18
**October-23**	14	163	8.59
**November-23**	5	163	3.07
**Unknown**	1	163	0.61

Supplementary table 12

RNA samples for sequencing; case distribution – Sample type used for extraction

District	n	Total	Percentage
**Peritoneal**	90	163	55.21
**Pleural**	24	163	14.72
**CSF**	43	163	26.38
**Nasal Swab**	1	163	0.61
**Tissue/LN[a] intestinal**	5	163	3.07

[a]LN – lymph node

Supplementary table 13

Successfully sequenced spike protein; case distribution – Sex

Sex	n	Total	Percentage
**Female**	25	63	39.68
**Male**	38	63	60.32

Supplementary table 14

Successfully sequenced spike protein; case distribution – Age stats (years). The ages of 14 cats are missing.

Mean_age	Median_age	Min_age	Max_age
4.44	3	1	16

Supplementary table 15

Successfully sequenced spike protein; case distribution – FIP form

Form	n	Total	Percentage
**Non-effusive**	5	63	7.94
**Neurological**	10	63	15.87
**Effusive**	48	63	76.19

Supplementary table 16

Successfully sequenced spike protein; case distribution – District

District	n	Total	Percentage
**Nicosia**	17	63	26.98
**Famagusta**	24	63	38.10
**Larnaca**	7	63	11.11
**Limassol**	9	63	14.29
**Paphos**	4	63	6.35
**UK**	2	63	3.17

Supplementary table 17

Successfully sequenced spike protein; case distribution – Collection dates

Date	n	Total	Percentage
**January-23**	3	63	4.76
**February-23**	8	63	12.70
**March-23**	20	63	31.75
**April-23**	12	63	19.05
**May-23**	5	63	7.94
**June-23**	3	63	4.76
**July-23**	3	63	4.76
**August-23**	5	63	7.94
**September-23**	1	63	1.59
**October-23**	2	63	3.17
**November-23**	2	63	3.17

Supplementary table 18

Successfully sequenced spike protein; case distribution – Sample type used for extraction

Form	n	Total	Percentage
**Peritoneal**	41	63	65.08
**Pleural**	8	63	12.70
**CSF**	10	63	15.87
**Tissue/LN[a] intestinal**	4	63	6.35

[a]LN – lymph node

Supplementary table 19

Preliminary primer sequences used during first amplifications of the FCoV-23 genome. Primers were designed either using primal scheme or through manual design following multi-sequence alignment of available FCoV whole genomes on NCBI with Mafft[1] (v7.490). This is not a finalised scheme and Supplementary table 20 primers should be used for sequencing FCoV-23.

Scheme location	Forward (5'-3')	Reverse (5'-3')	Specific target (where applicable)	Start (consensus genome)	End (consensus genome)	Expected length	Note
**28 to 32**	GACGCAGACTTCAGTGTTA	ACCATTATGCCATTRTARTA	Spike	19728	23341	3613	[a]
**23**	TGCCCAGCTGARATTGTTAARACAG	CATAGTTGTAAGYTCAAGACCACC	ORF1b region	16376	17426	1050	
**35**	TGTCTBAGTACTGGHTGYTGTGG	AGCATAGGGTCTACAAAATGCAAC	Orf3c/E/M region part 1	23592	24616	1024	
**36**	GATGGCATTGTKACAAYAACTGTCTT	ARCCGAACATTACATATCTGGAAACTT	Orf3c/E/M region part 2	24380	25489	1109	
**1**	GGACACCAACTCGAACTAAACGA	GTCCARTCACCDACACCACTACT		0	992	992	
**2**	GTAGCACCRCCAGTCAAGARRAAYTC	GTRGCRAARAATGCACTATCAAGRCC		801	1775	974	
**3**	GTGAARGCHTTYGATGTYTTCACACA	TACRGGCACACTATGCAGTCTTAA		1605	2676	1071	
**3 to 4**	TTGTCAAGCTTGTCAGTGT	ATTGAGCATCGTCTCCAA		1705	3726	2021	
**5 to 7**	TGGGCTGYTGCTGTYGAYGAACA	GTGTTTAAGYGCGAGAACTGMCTT		3438	6408	2970	
**8 to 9**	CAGTACMTYAACCTGTGHTRTC	CCACCRAAGCAAAAACCAGCT		6270	8135	1865	[b]
**9**	GGTAAGTGCATGACTTTYGATGC	CCACCRAAGCAAAAACCAGCT		7191	8135	944	
**9 to 11**	GGTAAGTGCATGACTTTYGATGC	ACCYACTGACCACARGTACCAGC		7191	9342	2151	
**10 to 14**	TGGTTATTAAGAAYGGTRTYGTTCAACC	TGYCTAAYCTTGGAAAGCACTTCA		7567	11363	3796	
**14 to 16**	GCTTACCATGTTGATAAGTCTTACTACAAA	AGACTTGCTCATGGTCCATAACG		10313	12501	2188	
**17**	TTAAACGAGTGCGGGGTTCTAG	GGYACACCATCWATRTGRACTTTACG		12282	13264	982	
**18 to 19**	ATGGGAATKACTTCATGTTTAGAR	CAGAAACAGCTTGAAAGATGT		12977	14352	1375	
**20**	ACAACYAGYGGTGATGGTACTACA	CCAAACACATTACCATTAGCACAGAG		14285	15316	1031	
**21 to 22**	GGCATGTGTGTTGTTTGTGGTT	TGTARATDACATAATCRTACTCACTACC		15050	16684	1634	
**23**	TGCCCAGCTGARATTGTTAARACAG	CATAGTTGTAAGYTCAAGACCACC	ORF1b region	16376	17426	1050	
**24 to 25**	TTTGCTATGCGTAATGTDAGAGCRTG	TGGCACTRAGYCCYTTCACAGC		17072	18835	1763	

**26 to 27**	AATRGYAAAGCMCTCCARAGT*	GARTTTCKCCAAAATATATAATTGGCATGC		Not present	20120	1648	[c]
**28 to 32**	GACGCAGACTTCAGTGTTA	ACCATTATGCCATTRTARTA	Spike	19728	23341	3613	[d]
**33**	GCCCTTAAYCTTGGYGCRCGTMT	ACRCATATTCCTGACCATGCCG		Not present	Not present		[e]
**34 to 35**	GCAGCACTTAAYGCBTATGYGTC	AGCATAGGGTCTACAAAATGCAAC		Not present	24616		[f]
**35**	TGTCTBAGTACTGGHTGYTGTGG	AGCATAGGGTCTACAAAATGCAAC	ORF3c/E/M region part 1	23592	24616	1024	
**36**	GATGGCATTGTKACAAYAACTGTCTT	ARCCGAACATTACATATCTGGAAACTT	ORF3c/E/M region part 2	24380	25489	1109	
**36 to 37**	GATGGCATTGTKACAAYAACTGTCTT	CTTATTACCTATTCCYTTGGGAACAA		24380	25407	1027	
**38**	CYGGTGATTACTCAACAGAAGCA	GTTTTGGCATCATCYTTGGCAGG		25779	26805	1026	
**38 to 40**	CYGGTGATTACTCAACAGAAGCA	ACATTTTAAACAATCACTAGATCCAGACG		25779	28167	2388	
**40**	CARCTTTTGARRCCAGACTGYC	ACATTTTAAACAATCACTAGATCCAGACG		27161	28167	1006	

[a]Also amplifies GHR, deletion variant; shorter. [b]Poor performance. [c]Amplification works, however F primer was not incorporated into the draft genome possibly due to variation in the amplified viruses. [d] Also amplifies GHR, deletion variant shorter. [e] Poor performance. [f] Very poor performance

Supplementary table 20

Tiled amplicon scheme for the amplification of the FCoV-23 genome. Primers were designed using primal scheme[2] and manual design using a composite FCoV-23 genome generated using primers in supplementary table 20. Amplicon lengths are designed for 800 bp lengths with 80 bp overlap. We recommend to first assess presence of full-length (FL) spike or domain 0 deletion using primers 36A-F and 37A-R.

Primer number	Forward (5'-3')	Location (FL genome)	LONG Pool	SHORT Pool	x-fold in Pool
**1-F**	GGACACCAACTCGAACTAAACGA		1	1	0.5
**1-R**	ARTTCTTCTTGACTGGTGGTGC		1	1	0.5
**2-F**	ACGGARTAAGTGATCTTAAACCTGTTCT		2	2	0.5
**2-R**	GAAGTCGTTCAATTCGCCTTCAAA		2	2	0.5
**3-F**	TCTTTGCTAAYAGTGTGCTCCAA		1	1	1
**3-R**	AAGCCATATCACCAAYAAYGACA		1	1	1
**4-F**	GCRCTTGTYAAGCTTGTCAGTG		2	2	1
**4A-F**	TGACACCACAGAGGACTGAGGC		2	2	1
**4-R**	AACYTTAAYACCATTACCTAGC		2	2	1
**4A-R**	TCCARTTCCAGCAGAAGGTCWG		2	2	1
**5-F**	CCAGTKTGTCTTAAAAACCATGTYGG		1	1	1
**5-R**	GTACCAATGACYTTTTCAAGCACC		1	1	1
**6-F**	AGAGATASAACCYGTTACACGTGTC		2	2	1
**6-R**	TCGTCTCCAAAAGRTATTCTGCATC		2	2	1
**7-F**	GATGAACAGGAAKCTGAACAACC		1	1	1
**7A-F**	GAYCCATGGGCTGCTGCTGTT		1	1	1
**7-R**	TCCKTGGTARAATGARACTTTTCCC		1	1	1
**7A-R**	CTTTGACGTTCTTCTGCTACRCAC		1	1	1
**8-F**	TGGATGGTATGGGAATTAAACCTCG		2	2	1
**8-R**	CACACTWGGYAACTGGTTAGT		2	2	1
**9-F**	TGTCTTYGTTTACACTGACCARGAG		1	1	1
**9-R**	CGTARTAGGTGTAATGACCACGYG		1	1	1
**10-F**	GCTTGKTGAKTTGATGTCRAGTG		2	2	2
**10-R**	ACTAGTTTTGCATARCGCCA		2	2	2
**11-F**	GCTGACGTRTTCTTTATGRCTGG		1	1	1
**11-R**	GCTTTTTRCAGAGTYTACCAGTACC		1	1	1
**12-F**	TCATGGGATTAYAAGTCAGACCC		2	2	1
**12-R**	TTGGCATTRAARAGTGCTCCCT		2	2	1
**13-F**	CARGAAGTGCTTAAGACTATGTTYC		1	1	1
**13A-F**	TTCACGGCATATGACTATGATG		1	1	1
**13B-F**	GAAGTATAGTAGTCAGGAAGTGC		1	1	2
**13-R**	CCATGTAATCATARCCCTCWGCAG		1	1	2
**13A-R**	CAAATGGTTGAACAACACCGTTC		1	1	2
**14-F**	CAGATGAAGATYTGCCKTATGAGCG		2	2	2
**14-R**	CCATCYCCAAACTCTTTATCATAGACA		2	2	2
**14A-R**	TCAATACACTCTCCGACTCTGC		2	2	2
**15-F**	TGAGGGTGCTAAGCTTTACAGTG		1	1	0.5
**15-R**	ATCAGCCTCTCCCATMGAACCA		1	1	0.5
**16-F**	TCYCTACCATCACTATTCAAACTTAARGT		2	2	1

Primer number	Forward (5'-3')	Location (FL genome)	LONG Pool	SHORT Pool	x-fold in Pool
**16-R**	GAGAGCCGTTACCTAATTCTAGATGG		2	2	1
**17-F**	GTCTGTGAAACCAGGTGAGAGTT		1	1	0.5
**17-R**	AACAACATTTTATGCTTAATTCCAACGAC		1	1	0.5
**18-F**	ACGCCTACTGAAGTCATAAGGC		2	2	0.5
**18-R**	ACTGTGAACTGGTAAACACCACA		2	2	0.5
**19-F**	CAAAGGACTGGTTTGTTGTTTTTGC		1	1	0.5
**19-R**	GCGTGCCTCTTTGTACATGCYA		1	1	0.5
**20-F**	TTGCCTAGCTGGATTGCCTATG		2	2	1
**20-R**	TATTTAACYTCAGGACCATTARCRCC		2	2	1
**21-F**	TGCTTATGGTAGYGGTAAAGCGC		1	1	0.5
**21-R**	AGCTCTACTAACATGGTCTGGATC		1	1	0.5
**22-F**	GWGGTATGCAGCCAGTTAMTAAYT		2	2	1
**22-R**	ACTGGRTCAAACCAATCCTTRTT		2	2	1
**23-F**	TGYAGTTGCTGAACAYGACTT		1	1	1
**23-R**	GKAGATCTGTCATRGTCAACTTCA		1	1	1
**24-F**	CATTGTGCYAATTTTAACACRYT		2	2	0.5
**24A-F**	CTGCATTTGGACCTCTTGTACGTA		2	2	0.5
**24-R**	CTGTCTCGTYGTCATTGTKGA		2	2	0.5
**24A-R**	TCCCACAGTGCGAGCTCTAGAC		2	2	0.5
**25-F**	TTTGGTAAGGCAAGACTTTACTATGAGA		1	1	1
**25-R**	TCTGCTACATARCCAAGATCWGCA		1	1	1
**26-F**	GCTTTTAGGAGTRGATTCAAACAC		2	2	1
**26A-F**	ACAGCGCAAGATATATGACAATTG		2	2	1
**26-R**	AGCTACACACATAWGGYGTAATAGAC		2	2	1
**26A-R**	GTAACATCATTAACAGTACAACCATT		2	2	1
**27-F**	CAAAACACCCTAARCCTGCWTATCAA		1	1	0.5
**27-R**	GAGTCACTACCATATTCTGACTGCTC		1	1	0.5
**28-F**	TGTGAAAGCWAAGGAGGARTCTGT		2	2	1
**28-R**	AARGTTCTAGGWGCTGGRAGTT		2	2	1
**29-F**	AGGATAATACCTCAAAGAATCAGAGTTGA		1	1	1
**29-R**	ACCACAAGTTTCAGGTTTTGCYTG		1	1	1
**30-F**	ACTCGGCKCAAGGTAGTGAGTA		2	2	0.5
**30-R**	CCATTTTGCCGCAMTCACATTT		2	2	0.5
**31-F**	TCATGAGGAGRGGTCAAYCYT		1	1	1
**31-R**	GCATGATTGTTAACATACAACGCAC		1	1	1
**32-F**	AAYAATGTTAGATGTCTGGAGTAYGA		2	2	1
**32-R**	AGTTTGAGAAAGGACAGTCCGC		2	2	1
**33-F**	AGGAACGGACCTACTGACAAGT		1	1	0.5
**33-R**	TCGTCCAAGAGTATGTCCATATAAGTG		1	1	0.5
**34-F**	TGGTTTTGAACACGTTGTATTTGGA		2	2	0.5
**34-R**	ATCACCTGTAACACTGAAGTCTGC		2	2	0.5
**35-F**	GCTCCTGGTAGTACTGTCYTAAGA		1	1	1
**35A-F**	GTCAAGTACACTCAGTTGTGTC		1	-	1
**35-R**	CACCAACAACYACASTTCCTTCT		1	-	1
**35A-R**	CGTTGCCATCTAATTGTGTTACG		1	-	1
**36-F**	GGCAARYTACTAAACTTTGGTAACC		2	2	1
**36A-F**	CTAAGGAAGGGTAAGTTGCTCA		2	2	1
**36-R**	CCAGTGCAATRTTCATAATCYTCACA		2	1	1

Primer number	Forward (5'-3')	Location (FL genome)	LONG Pool	SHORT Pool	x-fold in Pool
**36A-R**	CCATCAGGTATGTAACCTCCC		2	1	1
**37-F**	GTGGAATGATGAMYTTGTTACAGC		1	-	1
**37A-F**	ATACCCACGGACAATGGAACGA		1	-	1
**37-R**	AGGAAATGTGCTAAAGAAATTGTAACCA		1	2	1
**37A-R**	CCTTTACACTAGGTGGTAATGTTC		1	2	1
**38-F**	ACAGTGMGTGAGTCTAGTTYTTACA		2	1	1
**38-R**	CARTTAGCACCAACAGGAYKCA		2	1	1
**39-F**	ACGTGTATTGCATTCGTTCTAATCAA		1	2	0.5
**39-R**	GTATCGTGACATTACCRGTGCT		1	2	0.5
**40-F**	GTRACRCCATGTGATGTAAGCGC		2	1	2
**40-R**	TGCCATTGTAATATTGAGCACAMAC		2	1	2
**41-F**	GTGACATCTGGYTTAGGTACAGTCG		1	2	1
**41-R**	AACRAGTCCRAAAGTGYGATCG		1	2	1
**42-F**	ACWAGCAGAGGTTAGGGCTAGT		2	1	0.5
**42-R**	TCTACTGAAAAGAGAATGACAACAGCT		2	1	0.5
**43-F**	GTCTYAGTACTGGYTGTTGTGG		1	2	1
**43-R**	GTTRTTGTMACAATGCCATCTATGT		1	2	1
**44-F**	ACCACATGTTAATACCATAGTACAACAAC		2	1	0.5
**44-R**	CATGGCGTGCAGGTAGTACTAT		2	1	0.5
**45-F**	CGTGTCTATGATGTTTCCTAGGGC		1	2	0.5
**45-R**	TGTAGGTRTGCCATCAAGGGGT		1	2	0.5
**46-F**	TCGGCTTTAGTGTTGCAGGTG		2	1	0.5
**46-R**	AGTAGAAGAACCACCTTTCAGGAAG		2	1	0.5
**47-F**	CCCATTACCCTCGAAACAGGATC		1	2	0.5
**47-R**	GTRAGYGTGACTTTCACYTGATC		1	2	0.5
**48-F**	ATGCCAACAAACACASCTGG		2	1	0.5
**48-R**	CACACWAGGAYTACARCAATCATG		2	1	0.5
**49-F**	CCTGCTATACATTGTTAGGTGC		1	2	1
**49-R**	ACATTTTAAACAATCACTAGATCCAGACG		1	2	1

Supplementary table 21

Results of recombination analysis. Several tools run using RDP5 and using a multi sequence alignment between the assembled FCoV-23 genome 2-C11 Re 10276 (PQ133182), a pCCoVII genome (KP981644.1), an FCoVII genome (LC742526.1) and an FCoVI genome (MT239440.1) were used to analyse recombination.

Tool used via RDP5	P- values
**RDP5**[3]	4e-30 $x10^{-300}$
**GENECONV**	4e-30 $x10^{-300}$
**BootScan**[4]	8.624 $x10^{-269}$
**MaxChi**[5]	1.200 $x10^{-199}$
**Chimaera**[6]	2.948 $x10^{-64}$
**SiScan**[7]	8.209 $x10^{-93}$
**3Seq**[8]	4e-30 $x10^{-300}$

Supplementary table 22

Results of recombination analysis. Several tools run using RDP5 and using a multi sequence alignment between the assembled domain 0-deletion FCoV-23 genome 2-F12 BW 11350 (PQ133177), a pCCoVII genome (KP981644.1), an FCoVII genome (LC742526.1) and an FCoVI genome (MT239440.1) were used to analyse recombination.

Tool used via RDP5	P- values
**RDP5**[3]	2.000 $x10^{-299}$
**GENECONV**	2.000 $x10^{-299}$
**BootScan**[4]	4.414 $x10^{-21}$
**MaxChi**[5]	6.000 $x10^{-199}$
**Chimaera**[6]	6.000 $x10^{-199}$
**SiScan**[7]	9.679 $x10^{-87}$
**3Seq**[8]	2.000 $x10^{-299}$

Supplementary table 23

Comparison of FCoV-23 Spike-2 with determinant mutations. The consensus FCoV-23 sequence was compared with key sequence features that were identified in Zehr *et al.* [9] as positively associated with the FECV biotype. Association with biotype was assessed whether mutations had been previously more likely been associated with one of the biotypes. Amino acid positions below the line are after the recombination breakpoint between pCCoV and FCoV-1.

Position in FCoV-23	Amino acid sequence in FCoV-23	Amino acid composition at site associated with biotype
**534**	V	Marginally tentative FIPV
**596**	Q	Marginally tentative FIPV
**1404**	L	**New mutation**
**1405**	V	Marginally tentative FIPV
**1416**	L	Marginally tentative FECV
**1434**	L	Marginally tentative FECV

Supplementary table 24

Comparison of FCoV-23 Orf3a,b, and c with determinant mutations. The consensus FCoV-23 sequence was compared with key sequence features that were identified in Zehr *et al.* [9] as positively associated with the FECV biotype. Association with biotype was assessed whether mutations had been previously more likely been associated with one of the biotypes. Sites could previously not be "statistically associated uniquely with one phenotype"[9].

Protein	Position in FCoV-23	Amino acid sequence in FCoV-23	Amino acid composition at site associated with biotype
**Orf3a**	**30**	L	No indication
**Orf3a**	**32**	N	No indication
**Orf3a**	**47**	E	No indication
**Orf3a**	**58**	Q	No indication
**Orf3a**	**61-62 gap**	IE	No indication
**Orf3a**	**64**	S	No indication
**Orf3a**	**65**	S	**New mutation**
**Orf3b**	**2**	R	**New mutation**
**Orf3b**	**64**	K	No indication
**Orf3b**	**71**	A	No indication
**Orf3c**	**11**	S	No indication
**Orf3c**	**71**	G	No indication
**Orf3c**	**72**	V	No indication
**Orf3c**	**159**	M	No indication
**Orf3c**	**165**	T	No indication
**Orf3c**	**175**	G	No indication

Supplementary table 25

Comparison of FCoV-23 Orf7b with determinant mutations. The consensus FCoV-23 sequence was compared with key sequence features that were identified in Zehr *et al.* [9] as positively associated with the FECV biotype. Association with biotype was assessed whether mutations had been previously more likely been associated with one of the biotypes.

Position in FCoV-23	Amino acid sequence in FCoV-23	Amino acid composition at site associated with biotype
5	V	Marginally tentative FIPV
11	L	No indication
12	A	No indication
19	D	No indication
25	H	No indication
36	Q	No indication
39	V	No indication
41	H	No indication
48	H	No indication
50	I	No indication
63	S	No indication
68	N	No indication
82	I	No indication
89	S	No indication
106	N	No indication
107	Q	No indication
129	T	No indication
131	F	No indication
139	T	No indication
140	Q	No indication
145	R	No indication
147	F	No indication
149	H	No indication
152	S	No indication
159	**I**	**New mutation**
160	H	No indication
167	Y	No indication
168	C	No indication
170	H	No indication
172	L	No indication
187	K	No indication
190	R	No indication
191	S	No indication
194	V	No indication
198	L	No indication
199	N	No indication
200	Q	No indication
202	H	No indication
203	**H**	**New mutation**
204	T	No indication

Supplementary table 26

Reposited full-genome FCoV-23 sequences from this study.

GB Number	Isolate
**PQ133176**	1-F11 Gi 6590
**PQ133177**	2-F12 BW 11350
**PQ133178**	2-A6 Ge 7353
**PQ133179**	2-A9 Do 9045
**PQ133180**	2-B6 Lu 7456
**PQ133181**	2-C8 Wi 8299
**PQ133182**	2-C11 Re 10276
**PQ133183**	2-D4 An 7030
**PQ133184**	2-D8 Ta 8364
**PQ133185**	2-D9 Ni 9183
**PQ133186**	2-E6 Bi 7543
**PQ133187**	2-E8 Ru 8415
**PQ133188**	2-E9 Mi 9160
**PQ133189**	2-F3 Je 6971
**PQ133190**	2-F9 Fe 9265
**PQ133191**	2-G2 Ka 6882
**PQ133192**	2-H6 Ka 7790
**PQ133193**	2-H7 Ja 8138
**PQ133194**	2-H8 So 8842
**PQ133195**	2-H9 Mo 9241

References

1. Katoh, K., Misawa, K., Kuma, K. & Miyata, T. MAFFT: a novel method for rapid multiple sequence alignment based on fast Fourier transform. *Nucleic Acids Res* **30**, 3059-3066 (2002). https://doi.org/10.1093/nar/gkf436
2. Quick, J. *et al.* Multiplex PCR method for MinION and Illumina sequencing of Zika and other virus genomes directly from clinical samples. *Nature Protocols* **12**, 1261-1276 (2017). https://doi.org/10.1038/nprot.2017.066
3. Martin, D. P. *et al.* RDP5: a computer program for analyzing recombination in, and removing signals of recombination from, nucleotide sequence datasets. *Virus Evolution* **7** (2020). https://doi.org/10.1093/ve/veaa087
4. Martin, D. P., Posada, D., Crandall, K. A. & Williamson, C. A modified bootscan algorithm for automated identification of recombinant sequences and recombination breakpoints. *AIDS Res Hum Retroviruses* **21**, 98-102 (2005). https://doi.org/10.1089/aid.2005.21.98
5. Smith, J. M. Analyzing the mosaic structure of genes. *J Mol Evol* **34**, 126-129 (1992). https://doi.org/10.1007/bf00182389
6. Posada, D. & Crandall, K. A. Evaluation of methods for detecting recombination from DNA sequences: Computer simulations. *Proceedings of the National Academy of Sciences* **98**, 13757-13762 (2001). https://doi.org/10.1073/pnas.241370698
7. Gibbs, M. J., Armstrong, J. S. & Gibbs, A. J. Sister-Scanning: a Monte Carlo procedure for assessing signals in recombinant sequences. *Bioinformatics* **16**, 573-582 (2000). https://doi.org/10.1093/bioinformatics/16.7.573
8. Lam, H. M., Ratmann, O. & Boni, M. F. Improved Algorithmic Complexity for the 3SEQ Recombination Detection Algorithm. *Mol Biol Evol* **35**, 247-251 (2018). https://doi.org/10.1093/molbev/msx263
9. Zehr, J. D. *et al.* Natural selection differences detected in key protein domains between non-pathogenic and pathogenic feline coronavirus phenotypes. *Virus Evol* **9**, vead019 (2023). https://doi.org/10.1093/ve/vead019
